# Special Issue: “Yeast as a Model System to Study Human Diseases”

**DOI:** 10.3390/ijms26188919

**Published:** 2025-09-12

**Authors:** Nicoletta Guaragnella, Belém Sampaio-Marques, Tiziana Cervelli

**Affiliations:** 1 Department of Biosciences, Biotechnologies and Environment, University of Bari “Aldo Moro”, 70125 Bari, Italy; nicoletta.guaragnella@uniba.it; 2 Life and Health Sciences Research Institute (ICVS), School of Medicine, University of Minho, Campus de Gualtar, 4710-057 Braga, Portugal; mbmarques@med.uminho.pt; 3 ICVS/3B’s—PT Government Associate Laboratory, 4710-057 Braga, Portugal; 4 Institute of Clinical Physiology, CNR, 56124 Pisa, Italy

## 1. Introduction

Yeast is a well-known eukaryote that has been fundamental in the discovery of principles governing cellular life. Despite its phylogenetic distance from humans, budding yeast shares more than 2000 genes (approximately 30% of its genome) with humans [1]. In addition, 45% of its genome is replaceable with a human gene [2]. Studies on *Saccharomyces cerevisiae* have led to the discovery of key regulators of the cell cycle, mechanisms by which chromosomes are protected by telomeres and the enzyme telomerase, and processes that control autophagy [3]. Yeast has been instrumental in understanding how protein misfolding can lead to neurodegenerative diseases and how mutations in mismatch repair genes, such as *MSH2* and *MLH1*, can cause hereditary non-polyposis colorectal cancer [4]. Furthermore, yeast models have proven valuable for drug screening and the functional characterization of disease-associated genes identified through human genomics [5].

Taken together, this body of evidence supports the use of yeast as a robust system to elucidate the function of many human genes. However, the emergence of CRISPR/Cas9 technology has significantly facilitated genome editing in human cells and animal models, raising a critical question for yeast researchers: Can yeast remain a valid model system for biomedical studies?

This Special Issue clearly demonstrates that yeast continues to be a powerful model organism, offering valuable tools for both fundamental and applied biological research. The collected papers illustrate recent advances across various fields of cellular biology, each addressing important unresolved questions. We have grouped the contributions into three thematic sections: (1) Functional implications of human genetic variants; (2) Yeast as a sensing and screening/predictive platform for biomedical applications and drug discovery; and (3) Yeast models to unravel protein misfolding, neurodegeneration, and host–pathogen interactions.

## 2. Functional Implications of Human Genetic Variants

Establishing the connection between genetic variants and their impact on protein function remains a significant unmet challenge in the post-genomic era. Yeast has proven to be a powerful model system for investigating the functional consequences of protein variants. Notable examples include the characterization of p53 and PTEN variants using yeast-based assays [6,7]. The review article “Homo cerevisiae” by Laval et al. emphasizes the utility of yeast platforms in the high-throughput variant analysis, interactome mapping, and modeling of protein–protein interactions—key approaches that support the advancement of precision medicine (contribution 1). An illustrative example of the yeast-based analysis of human gene variants is provided by Galli et al., who used *S. cerevisiae* to investigate BRCA1 ∆11 splicing variants (contribution 2). *BRCA1* is a tumor suppressor gene frequently mutated in cancers, particularly breast and ovarian cancers. Exon 11, which accounts for 65% of the BRCA1 protein, does not correspond to a specific domain but interacts with numerous proteins involved in DNA repair, cell cycle regulation, and transcriptional control [8]. The authors had previously demonstrated the feasibility of using yeast to assess the functional impact of BRCA1 missense variants [9]. In their current study, they focus on the activity of ∆11 splicing variants and show that these alterations impair BRCA1 function similarly to pathogenic missense mutations, suggesting a potential role in tumorigenesis (contribution 2).

Yeast is a well-established model organism for investigating the molecular mechanisms underlying pathogenic mutations in human mitochondrial genes [10]. One key enzyme located in the inner mitochondrial membrane is the cytochrome c oxidase (COX) holoenzyme, which consists of multiple subunits. The proper assembly of these subunits relies on a growing set of proteins known as COX assembly factors. The review by Caron-Gordon highlights how the high degree of conservation of these assembly factors between yeast and humans has enabled researchers to study the functional impact of patient-derived variants associated with COX deficiencies (contribution 3).

## 3. Yeast as a Sensing and Screening/Predictive Platform for Biomedical Applications and Drug Discovery

The use of yeast as a biosensing and screening/predictive platform for medical and industrial applications is well established [11,12]. Our Special Issue includes four contributions on this topic. The research article by Mulvihill et al. presents a humanized yeast biosensor for the screening of cannabinoid compounds. Cannabinoids are used in the treatment of chronic pain, epilepsy, and psychiatric disorders and there is growing demand for next-generation cannabinoid medicines. By combining synthetic biology approaches that target receptor trafficking and membrane composition, the authors screened large libraries of compounds, characterized known effectors, and discovered novel biological functions. Their study paves the way for adapting yeast sensors for pharmaceutical drug development (contribution 4).

Among the widely used chemotherapeutic drugs is 5-Fluorouracil (5-FU), which interferes with nucleotide synthesis, essential for DNA replication and repair. However, resistance to 5-FU remains a significant obstacle to its effectiveness. Lim et al. screened a collection of *Schizosaccharomyces pombe* (fission yeast) null mutants and identified biological processes such as membrane transport, chromosome segregation, and mitochondrial oxidative phosphorylation as potential targets to increase drug sensitivity. Chromatin remodeling was also highlighted as a key factor influencing 5-FU resistance (contribution 5).

Understanding the molecular mechanisms behind physiological and pathological processes is crucial for identifying drug targets, their modes of action, and potential side effects. In this regard, yeast might offer another valuable opportunity, as reported in the review article by Bhadra and Xu on the Tel2-Tti1-Tti2 (TTT) complex. This complex is involved in the co-chaperone mechanism for the proper folding and stability of a group of proteins named phosphatidylinositol 3-kinase-related kinases (PIKKs). The complex works in distinctive pathways such as genome stability, telomere maintenance, and life span. Despite some controversy, the TTT complex appears to be largely conserved between yeast and humans. This is especially attractive for the drug Ivermectin, which inhibits Tel2, and could be the driver for a new wave of research into the TTT complex for the benefits of patients with cancer or other diseases (contribution 6).

The study of metabolic context represents a critical issue in cellular physiopathology and metabolic disorders. The yeast model has the power of easy genetic manipulation for the characterization of metabolic signals and their assessment upon a variety of environmental conditions. The research article by Groth et al. is an example of the elucidation of the molecular basis for the complex interplay between Nicotinamide adenine dinucleotide (NAD+) metabolism and phosphate sensing. By combining genetic and gene expression studies, the authors were able to identify different regulators and integration elements of NAD+ metabolism and to clarify different levels of interactions within and between signaling networks in the cell (contribution 7).

## 4. Yeast Models to Unravel Protein Misfolding, Neurodegeneration, and Host–Pathogen Interactions

*S. cerevisiae* and other yeast species have gained renewed recognition as powerful model systems for studying fundamental mechanisms underlying human diseases [13,14]. This Special Issue showcases the diverse applications of yeast in elucidating the fundamental mechanisms underlying protein misfolding and toxicity. The study by Stanford et al. revisits the mechanism by which overexpression of the molecular chaperone Hsp104 alleviates the [PSI^+^] prion in yeast. Through a series of elegant experiments, the authors provide compelling evidence that prion curing does not rely on the asymmetric segregation of seeds during cell division, as previously proposed. Instead, they demonstrate that Hsp104 overexpression leads to the direct dissolution of prion seeds, even in non-dividing cells, supporting a model driven by the chaperone’s trimming activity. These findings help resolve a long-standing debate in the prion biology field and highlight the nuanced regulatory roles of protein disaggregases in prion clearance (contribution 8).

The transactivating response (TAR) element DNA-binding of 43 kDa (TDP-43) is an aggregation-prone nuclear ribonucleoprotein (hnRNP) capable of translocating to the cytoplasm to assemble into cytoplasmic RNP granules. Pathological inclusions of cytoplasmic TDP-43 aggregates, commonly observed in the neurons of patients with ALS (amyotrophic lateral sclerosis) and frontotemporal lobar degeneration (FTLD), also forms in yeast. This Special Issue includes two studies focused on TDP-43 toxicity and potential suppressors. Peggion et al. find that nucleolin (NCL) can mitigate TDP-43-induced cytotoxicity. Using both yeast and human cell models, they demonstrate that the N-terminal and the central RNA-recognition regions of NCL are essential for its protective function, whereas the disordered C-terminal tail is dispensable (contribution 9). Park et al. link TDP-43 toxicity to the inhibition of autophagy (contribution 10). Autophagy is inhibited by rapamycin complex 1 (TORC1) [15]. The authors demonstrate that TDP-43 contrasts the aggregation of TORC1 in the TORC1-organized inhibited domain (TOROID), formation that is responsible for the control of TORC1-mediated autophagy inhibition (contribution 10).

The investigation of mitochondrial morphology and its impact on human diseases and microbial engineering has attracted considerable attention over the past decade. The “shape matter” review by Kichuk and Alvos explores how the mitochondrial morphology studied in yeast increases our understanding of the pathophysiology of neurodegenerative diseases and mitochondrial diseases caused by mutations in genes involved in mitochondrial fusion. The researchers also highlight how mitochondrial morphology engineering has been harnessed for chemical production (contribution 11).

A thorough understanding of host–pathogen interactions is crucial for developing effective therapeutic strategies against infections. This Special Issue features two articles that explore this important topic. Wevers et al. used *S. pombe* in a functional screen to identify *Chlamydia pneumoniae* proteins that alter the host microtubule cytoskeleton, a structure traditionally overlooked in chlamydial pathogenesis. The authors report that 13 proteins, mostly predicted to be inclusion membrane proteins, cause significant changes in microtubule organization and increase sensitivity to microtubule-destabilizing agents in yeast. The detailed characterization of CPn0443 as a microtubule-binding protein offers new insights into how *C. pneumoniae* manipulates host cell architecture, showcasing the value of yeast for dissecting host–pathogen interactions (contribution 12). The review by Fonseca-Fernández et al. offers a comprehensive overview of the evolution and application of genome-scale metabolic models (GEMs) applied to the study of fungal pathogens. GEMs are computational tools that simulate an organism’s entire metabolic network based on its genome. They enable the system-level analysis of metabolism and are instrumental in identifying new therapeutic targets, combating antifungal resistance, and understanding host–pathogen interactions. The authors show the potential applications of GEMs in the study of virulence and resistance of fungal pathogens, as well as the limitations of this approach (contribution 13).

## 5. Conclusions

This Special Issue reinforces the enduring relevance of yeast as a model organism in biomedical research. The contributions presented here underscore yeast’s versatility across a broad spectrum of applications, from variant interpretation and drug screening to uncovering mechanisms of protein misfolding, neurodegeneration, and host–pathogen interactions. As biomedical research increasingly depends on integrative, system-level approaches, yeast remains a powerful ally in deciphering the molecular underpinnings of human disease and driving innovation in therapeutic development.
